# *Drosophila* Relish Activating *lncRNA-CR33942* Transcription Facilitates Antimicrobial Peptide Expression in Imd Innate Immune Response

**DOI:** 10.3389/fimmu.2022.905899

**Published:** 2022-06-02

**Authors:** Hongjian Zhou, Shanshan Wu, Li Liu, Ruimin Li, Ping Jin, Shengjie Li

**Affiliations:** ^1^Laboratory for Comparative Genomics and Bioinformatics & Jiangsu Key Laboratory for Biodiversity and Biotechnology, College of Life Science, Nanjing Normal University, Nanjing, China; ^2^College of Biology and Food Engineering, Anyang Institute of Technology, Anyang, China; ^3^Jiangsu Provincial Key Construction Laboratory of Special Biomass Byproduct Resource Utilization, School of Food Science, Nanjing Xiaozhuang University, Nanjing, China

**Keywords:** *lncRNA-CR33942*, Relish, Imd signaling pathway, *Drosophila melanogaster*, transcriptional regulation, long noncoding RNA, survival

## Abstract

Long noncoding RNAs (lncRNAs) are an emerging class of regulators that play crucial roles in regulating the strength and duration of innate immunity. However, little is known about the regulation of *Drosophila* innate immunity-related lncRNAs. In this study, we first revealed that overexpression of *lncRNA-CR33942* could strengthen the expression of the Imd pathway antimicrobial peptide (AMP) genes *Diptericin* (*Dpt*) and *Attacin-A* (*AttA*) after infection, and vice versa. Secondly, RNA-seq analysis of *lncRNA-CR33942*-overexpressing flies post Gram-negative bacteria infection confirmed that *lncRNA-CR33942* positively regulated the *Drosophila* immune deficiency (Imd) pathway. Mechanistically, we found that *lncRNA-CR33942* interacts and enhances the binding of NF-κB transcription factor Relish to *Dpt* and *AttA* promoters, thereby facilitating *Dpt* and *AttA* expression. Relish could also directly promote *lncRNA-CR33942* transcription by binding to its promoter. Finally, rescue experiments and dynamic expression profiling post-infection demonstrated the vital role of the Relish/*lncRNA-CR33942*/AMP regulatory axis in enhancing Imd pathway and maintaining immune homeostasis. Our study elucidates novel mechanistic insights into the role of *lncRNA-CR33942* in activating *Drosophila* Imd pathway and the complex regulatory interaction during the innate immune response of animals.

## Introduction

Innate immunity plays the first and foremost role in the defense against pathogenic microorganisms (1). *Drosophila melanogaster* is a vital model for studying innate immunity because of the lack of highly specific adaptive immunity (2, 3). The *Drosophila* innate immune system comprises cellular immunity and humoral immunity which includes the production of many antimicrobial peptides (AMPs) (3, 4). The immune deficiency (Imd) signaling pathway is essential for Gram-negative bacterial invasion (5, 6). Once attacked by Gram-negative bacteria, the Imd pathway is activated and the downstream transcription factor *Relish* enters the nucleus to initiate the expression of AMPs (7). Although the major molecules of the Imd pathway have been identified, the complexity of immune regulation requires to explore more regulators to understand the immune homeostasis mechanism.

Dysregulation of immune homeostasis remarkably affects *Drosophila* survival and can lead to death (8, 9). Therefore, the intensity and duration of the immune response must be precisely regulated by many positive and negative regulators. For example, Akirin, Charon, sick, and STING can promote the Imd pathway by regulating the transcription factor Relish (10–13). In addition, some ubiquitin-related enzymes and peptidoglycan recognition proteins can positively regulate the Imd pathway (14–17). In contrast, some immune suppressors can restore immune homeostasis *via* preventing excessive activation of the Imd pathway (18–22). Our previous studies demonstrated that miR-9a, miR-981, and miR-277 could negatively regulate the Imd pathway by directly inhibiting the expression of *Diptericin* (*Dpt*), *imd*, and *Tab2* (23, 24). However, the regulatory mechanisms involved in maintaining immune homeostasis by emerging noncoding RNAs require further study.

Long noncoding RNAs (lncRNAs) are a class of RNAs of over 200 nucleotides that lack an open reading frames coding protein longer than 100 aa (25, 26).. lncRNAs exist widely in organisms, and their abundance is much higher than that of protein-coding RNAs (27). For example, the number of *Drosophila* lncRNA transcripts reached 42,848, and the number of lncRNA genes was 15,543 (28). To date, several studies have demonstrated that numerous lncRNAs participate in regulating a wide range of *Drosophila* biological processes, such as bristle formation (29, 30), embryo development (31–36), gonadal cell production (37, 38) and neuromuscular junctions (39, 40). A previous study has shown that lncRNA-VINR can defend against viruses by inducing AMPs (41). In addition, our previous studies demonstrated that *lncRNA-CR46018*, *lncRNA-CR11538* and *lncRNA-CR33942* could regulate *Drosophila* Toll innate immunity by interacting with the transcription factor Dif/Dorsal (42–44). Although some lncRNAs regulating *Drosophila* antiviral and Toll innate immunity have been discovered, it is largely unknown whether and how lncRNAs regulate the Imd signaling pathway against Gram-negative bacteria.

In this study, we first found that *Drosophila lncRNA-CR33942* can promote the Imd signaling pathway using RNA sequencing, and then, we quantified the expression levels of AMPs *Dpt* and *AttA* in *lncRNA-CR33942*-overexpressing flies, *lncRNA-CR33942* knockdown flies, and *lncRNA-CR33942* + *lncRNA-CR33942*-RNAi co-overexpressing flies after *Escherichia coli* infection. Second, using RIP-qPCR, ChIP-qPCR, and dual-luciferase reporter assays, we confirmed that *lncRNA-CR33942* interacts with the transcription factor Relish and strengthens the binding between Relish and the promoters of *Dpt* and *AttA*, thereby enhancing *Dpt* and *AttA* transcription. Third, we verified that the transcription factor Relish could also directly activate the transcription of *lncRNA-CR33942 via* ChIP and dual-luciferase reporter assays. Finally, the dynamic expression of *Dpt*, *AttA*, *Relish*, and *lncRNA-CR33942* in wild-type flies at different time points post-infection indicated the physiological function of this regulatory axis in the Imd immune pathway. In conclusion, our study discovered a novel Relish/lncRNA-CR33942/AMP regulatory axis, which plays a vital role in enhancing the immune response and maintaining immune homeostasis.

## Materials and Methods

### Fly Husbandry and Strains

The flies were raised in a standard corn flour/agar/yeast medium in a 25 ± 1°C incubator with a 12 h light/dark cycle. The fly stocks *w^1118^* (#3605), Tub-Gal80^ts^; TM2/TM6B (#7019), Tub-Gal4/TM3, Sb1, Ser1 (#5138), lncRNA-CR33942-RNAi (#30509), UAS-FLAG-Rel68 (#55777), and Relish-RNAi (#28943) were purchased from the Bloomington Drosophila Stock Center (Bloomington, IN, USA). The UAS-lncRNA-CR33942 fly stock was constructed in our laboratory previously (44). To eliminate false positives caused by overexpression, we also constructed *lncRNA-CR33942* + *lncRNA-CR33942*-RNAi co-overexpressing flies to reduce the overexpression of *lncRNA-CR33942*. To explore whether Relish regulates the Imd pathway through *lncRNA-CR33942*, Rel68 + *lncRNA-CR33942*-RNAi co-overexpressing flies were constructed. To overexpress or knockdown the corresponding gene in flies at a specific time, they were crossed with flies carrying Tub-Gal80^ts^ and incubated at 18°C. The adults were transferred to a 29°C incubator and cultured for over 24 h to overexpress or knockdown the genes.

### Sepsis and Survival Assays

Sepsis experiments were conducted on adult males aged 3–5 d. The *lncRNA-CR33942*-overexpressing, *lncRNA-CR33942*-RNAi, *lncRNA-CR33942* + *lncRNA-CR33942*-RNAi co-overexpressing, and control flies were infected with the Gram-negative bacterium *E. coli*. The infection experiment was performed *via* Nanoject instrument (Nanoliter, 2010; WPI, Sarasota, FL, USA). The glass capillary with full of *E. coli* suspension was inserted into the thorax and collecting the flies at the needed times for subsequent experiments. The survival assays post-infection indicate insufficient immune responses (45). The survival rate of ≥ 100 flies/group was monitored for 96-h after infection with *Enterobacter cloacae* concentrate.

### RNA Extraction and RT-qPCR

The total RNA of flies with different genotypes and treatments was extracted using RNA isolator total RNA extraction reagent (Vazyme Biotech Co. Ltd., Nanjing, China). And then, cDNA was obtained using a first-strand cDNA synthesis kit (Vazyme Biotech Co., Ltd., Nanjing, China). qPCR was performed using a BIO-RAD CFX Connect real-time PCR system (BIO-RAD, Hercules, California, USA) *via* AceQ SYBR Green Master Mix (Vazyme Biotech Co. Ltd., Nanjing, China). Our RT-qPCR cycling conditions were: step 1: 95°C for 5 minutes; step 2: 95°C for 10 seconds; step 3: 60°C for 30 seconds, then steps 2 and 3 were cycled for 40 times. The rp49 expression levels were used as the control to normalize other mRNAs. All the primers used for RT-qPCR are listed in **Supplementary Table 1**. All experiments were carried out in triplicate, and each biological sample was measured in triplicate by qPCR. The 2^-△△Ct^ method was used for data analysis (14). All qPCR data was showed as the mean ± SEM.

### Transcriptome Sequencing and Enrichment Analyses

RNA integrity was assessed with the RNA Nano 6000 Assay Kit for the Bioanalyzer 2100 system (Agilent Technologies, Santa Clara, CA, USA). Briefly, mRNA was purified using poly T oligo-attached magnetic beads and fragmentated to about 370–420 bp. Library fragments were purified, and PCR was performed with index (X) primers and universal PCR primers. The PCR products were purified, and the library quality was assessed using the Agilent Bioanalyzer 2100 system (Agilent Technologies, Santa Clara, CA, USA). According to the manufacturer’s instructions, index-coded samples were clustered on a cBot cluster generation system using the TruSeq PE cluster kit v3-cBot-HS (Illumina, San Diego, CA, USA). The reference genome index was built and paired-end clean reads were aligned to the reference genome using Hisat2 v2.0.5. FeatureCounts v.1.5.0-p3 enumerated the reads that mapped to each gene. Differential expression analysis was performed using the DESeq2 package of R v. 1.20.0 (R Core Team, Vienna, Austria). Genes with adjusted *P* < 0.05 were deemed differentially expressed genes (DEGs). And using the clusterProfiler package in R 4.0.3 (R Core Team, Vienna, Austria) for Gene ontology (GO) and Kyoto Encyclopedia of Genes and Genomes (KEGG) enrichment analyses on the DEGs. The local version of the GSEA tool (http://www.broadinstitute.org/gsea/index.jsp) was used for GSEA analysis.

### Prediction of Interaction Potential

The interaction potential between *lncRNA-CR33942* and Relish was predicted using RPISeq (RNA/protein interaction prediction tool, http://pridb.gdcb.iastate.edu/RPISeq/) (46), with known the interaction pair roX2 and msl-2 as the positive control.

### Construction of Recombinant Plasmid

To explore the effect of Relish on the *lncRNA-CR33942* promoter, we obtained the upstream 2-kb promoter sequence of *lncRNA-CR33942* from FlyBase (http://flybase.org) and cloned it into the pGL3-Basic plasmid. All the primers used for plasmid construction are listed in **Supplementary Table 1**. The pIEx4-Flag-Rel68, pGL3-Dpt-promoter, and pGL3-AttA-promoter plasmids were shared by Professor Xiaoqiang Yu (47). The pAc*-lncRNA-CR33942* plasmid was constructed as described previously (44).

### S2 Cell Culture and Transfection

*Drosophila* S2 cells were grown in a 28°C constant temperature incubator using SFX insect medium (HyClone Laboratories, Logan, UT, USA) with 10% (v/v) fetal bovine serum, 100 U/mL penicillin, and 100 μg/mL streptomycin (Invitrogen, Carlsbad, CA, USA). Transfection was performed using the X-treme gene HP transfection reagent (Roche Diagnostics, Basel, Switzerland) according to the manufacturer’s instructions. Briefly, S2 cells were transiently transfected with 200 μL of transfection complex containing 2000 ng plasmids in 6-mm plates (Corning, Corning, NY, USA) or 50 μL of transfection complex containing 500 ng plasmids in 24-well plates (Corning, Corning, NY, USA).

### RNA-Immunoprecipitation (RIP)

The detailed process of the RIP experiment followed this protocol (48). Briefly, approximately 3 × 10^7^ S2 cells transfected with Flag-Rel-N were lysed in radioimmunoprecipitation (RIPA) buffer (Beyotime Biotechnology, Shanghai, China) containing a protease inhibitor cocktail (Roche Diagnostics, Basel, Switzerland) and an RNase inhibitor (Thermo Fisher Scientific, Waltham, MA, USA) for 30 min on ice. The supernatants were pre-cleared for 1 h at 4°C using protein A agarose (Invitrogen, Carlsbad, CA, USA). After pre-clearing, anti-Flag-labelled or control anti-IgG antibodies (ABclonal Biotechnology Co. Ltd., Hubei, China) were separately added to the supernatants and the complexes were incubated at 4°C for 8-12 h. The next day, protein A agarose was joined and binding for 2 h. The beads were washed for five times using RIPA buffer. The remaining complexes were eluted using TE buffer with 1% (w/v) SDS. The eluted complexes of Flag-Rel-N and RNA were treated with protease K to separate the protein-bound RNA, and RNA was extracted and quantified using RT-qPCR.

### Chromatin Immunoprecipitation Sequencing (ChIP)-Seq and Transcription Factor Binding Site Analysis

ChIP-seq of *Relish* was obtained from the ENCODE project (https://www.encodeproject.org/). ChIP-seq peak analysis was performed using the ChIPseeker package in R (49). IGV 2.9.4 was used for peak visualization of ChIP-seq. The promoter sequences of *Dpt*, *AttA*, and *lncRNA-CR33942* were obtained from FlyBase (http://flybase.org/). In addition, the *Relish* motif was obtained in this study (50). The promoter sequences of *Dpt*, *AttA*, *lncRNA-CR33942* and *Relish* motifs were submitted to MEME (https://meme-suite.org/meme/tools/meme) and PROMO (http://alggen.lsi.upc.es/cgi-bin/promo_v3/promo/promoinit.cgi?dirDB=TF_8.3/). The RT-qPCR primers were designed based on the predicted binding sites.

### ChIP

ChIP experiments were performed following this paper (23). Briefly, S2 cells transfected with Flag-Rel-N were cross-linked using 1% (v/v) formaldehyde, lysed with RIPA and nuclear lysis buffers, and sonicated to shear to 200–800-bp fragments. ChIP incubation was performed using Dynabeads protein G (Thermo Fisher Scientific, Waltham, MA, USA) which was coated with anti-Flag or anti-IgG antibodies (ABclonal Biotechnology Co. Ltd., Hubei, China). After five times washings using different buffers, Flag-Rel-N-bound DNA was eluted and the cross-links between Rel-N and DNA were reversed at 65°C overnight. The eluted DNA fragments were purified for subsequent qPCR analysis. the primers of ChIP-qPCR were listed in **Supplementary Table 1**. All experiments were carried out in triplicate, and each biological sample was measured in triplicate by qPCR using the AMP promoters.

### Dual-Luciferase Reporter Experiment

To investigate the effects on the transcriptional regulation of *lncRNA-CR33942, Dpt*, and *AttA* promoters by *Relish* or *lncRNA-CR33942*, S2 cells were transfected using 50 μL of transfection complex containing pIEx4-Flag-Rel68, pGL3-Dpt-promoter, pGL3-AttA-promoter or pGL3-*lncRNA-CR33942*-promoter, pAc-empty or pAc-*lncRNA-CR33942*, and *Renilla* luciferase plasmid (pRL). pRL plasmids (Promega, Madison, WI, USA) were used for normalization and normalization. According to the manufacturer’s instructions, luciferase activity was measured using a Dual Luciferase Reporter Assay Kit (Vazyme Biotech Co. Ltd., Nanjing, China).

### Quantitation and Statistical Analysis

Experimental data were collected from three independent biological replicates and are presented as the mean ± SEM. Significant differences between values under different experimental conditions were analyzed using a two-tailed Student’s t-test. Fly survival analysis was performed using log-rank (Mantel-Cox) tests. Graphs were plotted using GraphPad Prism v. 8.3 (GraphPad Software, La Jolla, CA, USA) and RStudio (R Core Team, Vienna, Austria). Statistical significance was set at *P < 0.05*. *P < 0.05; **P < 0.01; ***P < 0.001; ns, not significantly different from the control.

## Results

### *LncRNA-CR33942* Is a Positive Regulator of the *Drosophila* Imd Signaling Pathway

**Figure 1:**
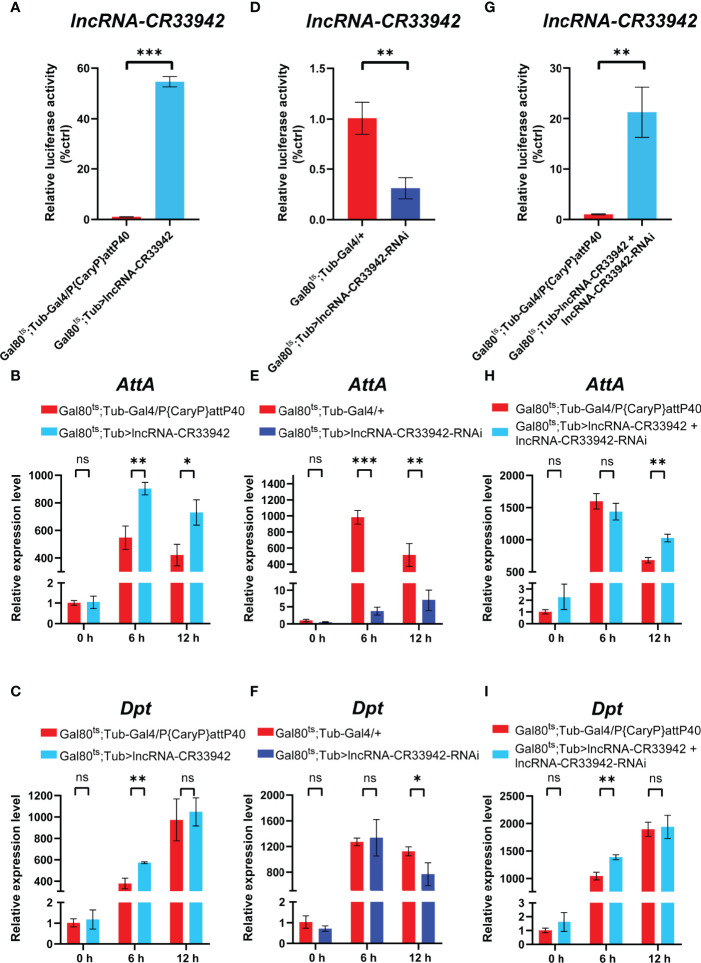
*lncRNA-CR33942* regulates Imd pathway AMPs after *Escherichia coli* infection. The expression levels of *lncRNA-CR33942* **(A, D, G)**, *AttA* **(B, E, H)** and *Dpt* **(C, F, I)** in *lncRNA-CR33942*-overexpressing flies, *lncRNA-CR33942*-RNAi flies, and *lncRNA-CR33942* + *lncRNA-CR33942*-RNAi co-overexpressing flies at different time points (0, 6, and 12 h) after *E. coli* infection. *P < 0.05; **P < 0.01; ***P < 0.001; ns, not significantly different from the control.

We demonstrated that *lncRNA-CR33942* could interact with Dif/Dorsal and facilitate AMP transcription, enhancing *Drosophila* Toll immune responses (44). Interestingly, *lncRNA-CR33942* may also regulate the Imd pathway through our previous RNA-seq in *lncRNA-CR33942-*overexpressing flies infected with *Micrococcus luteus*. To explore the effect of *lncRNA-CR33942* on the Imd pathway, we examined the expression levels of *Dpt* and *AttA*, two marker AMPs of the Imd pathway, in *lncRNA-CR33942*-overexpressing, *lncRNA-CR33942*-RNAi, and *lncRNA-CR33942* + *lncRNA-CR33942*-RNAi co-overexpressing flies at different time points (0, 6, and 12 h) after *E. coli* infection. Our results showed that the expression of *lncRNA-CR33942* in *lncRNA-CR33942*-overexpressing flies was approximately 60 fold than the control flies (**Figure 1**). Furthermore, the expression of *AttA* in *lncRNA-CR33942*-overexpressing flies was significantly upregulated compared to that in control flies at 6 and 12 h after *E. coli* infection, while the expression of *Dpt* was also significantly increased at 6 h post-infection (**Figure 1C**). In *lncRNA-CR33942*-RNAi flies with a 60% decrease in *lncRNA-CR33942* expression, *AttA* expression was dramatically inhibited post-infection, and *Dpt* expression was also markedly downregulated at 12 h post-infection (**Figures 1E, F**). Remarkably, the knockdown of *lncRNA-CR33942* seemed to block the induction of *AttA* from infection, and the expression level of *AttA* declined several hundred times compared to that in the control flies. To exclude the false-positive result of overexpression of *lncRNA-CR33942*, we constructed *lncRNA-CR33942* rescued flies (*lncRNA-CR33942 + lncRNA-CR33942-RNAi* co-overexpressing flies) with an approximately 20 fold upregulation of *lncRNA-CR33942* expression (**Figure 1**). The expression of *AttA* at 12 h post-infection and the expression of *Dpt* at 6 h post-infection were also significantly enhanced (**Figures 1I**). However, *lncRNA-CR33942* did not influence AMP expression under physiological conditions (0 h). Overall, these results indicate that *lncRNA-CR33942* can fine-tune AMP production in response to *E. coli* invasion, suggesting that it may regulate the *Drosophila* Imd pathway.

**Figure 2:**
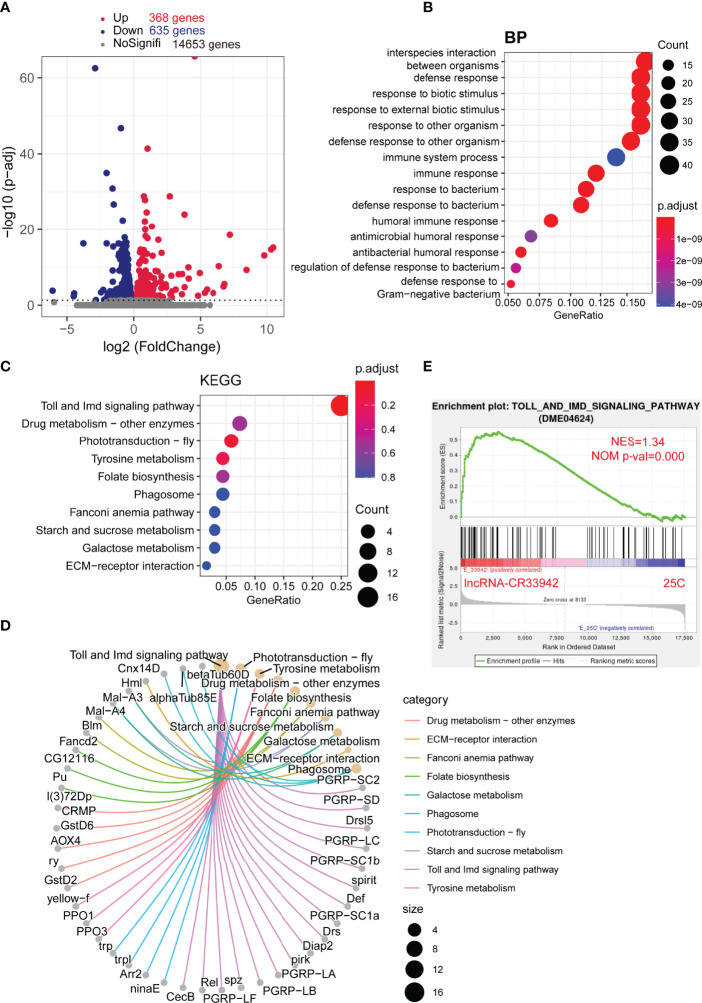
Enrichment analysis of DEGs in the *lncRNA-CR33942*-overexpressing flies after infection with *Escherichia coli*. **(A)** Volcano map shows DEGs between *lncRNA-CR33942*-overexpressing and control flies after *E. coli* infection. Red: upregulated DEGs (adjusted *P* < 0.05); blue: downregulated DEGs (adjusted *P* < 0.05). **(B)** Bubble chart shows biological process (BP) enrichment analysis of upregulated DEGs. **(C)** Bubble chart shows the KEGG pathway enrichment analysis of upregulated DEGs. **(D)** Network chart displays the KEGG pathway enrichment analysis and corresponding upregulated DEGs. **(E)** GSEA of the RNA-seq data between the *lncRNA-CR33942*-overexpressing and control flies.

To further confirm the regulatory function of *lncRNA-CR33942* in the *Drosophila* Imd pathway, we performed transcriptome sequencing of *lncRNA-CR33942*-overexpressing and control flies 12 h after *E. coli* infection. DEGs were identified with an adjusted *P* < 0.05. The sequencing results revealed 368 upregulated and 635 downregulated DEGs, while the remaining 14,653 genes were not differentially expressed (**Figure 2**). The enrichment and annotation of biological processes (BP) for the 368 upregulated DEGs mainly focused on defense, immunity, and response to stimuli (**Figure 2**). Remarkably, the upregulated DEGs were only significantly enriched in the Toll and Imd signaling pathways (**Figure 2**), and 13/16 DEGs were AMPs and peptidoglycan recognition proteins (PGRPs) (**Figure 2**). Considering that enrichment analysis with DEGs is biased, the overall pathway situation cannot be considered. We also used the GSEA algorithm to detect the expression of all the genes in the pathway (51). The results showed that the Toll and Imd signaling pathways were significantly enhanced in *lncRNA-CR33942*-overexpressing flies after infection with NES=1.34 and normal P value=0.000 (**Figure 2**). These results are consistent with the detection of AMPs, confirming that *lncRNA-CR33942* positively regulates the *Drosophila* Imd pathway.

### *LncRNA-CR33942* Promotes Relish Binding to the AMPs Promoter *via* Interaction

**Figure 3:**
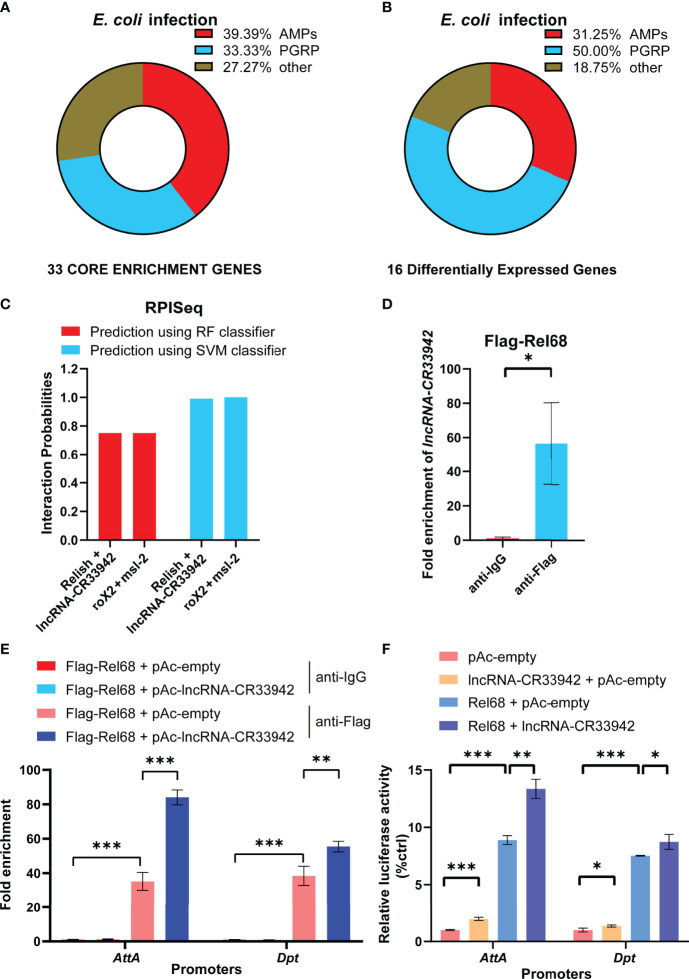
*lncRNA-CR33942* promotes Relish binding to the AMPs promoter *via* interaction. Classification analysis was performed on 33 core enriched genes from GSEA results **(A)** and 16 DEGs **(B)**. **(C)** Predicted scores for interaction potential of *lncRNA-CR33942* with Relish *via* RPISeq databases. **(D)** *lncRNA-CR33942* enrichment fold was measured by RIP-qPCR using anti-Flag antibody to immunoprecipitate overexpressed Flag-Rel68 in S2 cells. **(E)** ChIP-qPCR was performed on *lncRNA-CR33942*-overexpressing S2 cells with Flag-Rel68 overexpression normalized to control expression levels. **(F)** The dual-luciferase reporter assays were performed to detect the transcriptional activity of Rel68 on *Dpt* and *AttA* promoters with or without *lncRNA-CR33942* overexpression or only overexpressing *lncRNA-CR33942*. *P < 0.05; **P < 0.01; ***P < 0.001.

To explore how *lncRNA-CR33942* positively regulates the *Drosophila* Imd signaling pathway, we analyzed the results of GSEA and DEGs in the RNA-seq data. GSEA showed that 39.39% of AMPs and 33.33% of PGRPs were core enriched in the Toll and Imd signaling pathways (**Figure 3**). Similarly, DEGs of the Toll and Imd signaling pathways contained 31.25% AMPs and 50% PGRPs (**Figure 3**). AMPs and PGRPs were mainly transcribed by NF-κB proteins (52–55). Considering that *lncRNA-CR33942* functions in the nucleus and interacts with Dif/Dorsal to promote transcription, we speculated that *lncRNA-CR33942* interacts with Relish to regulate AMP transcription (44). To test this hypothesis, the RPISeq website was used to predict the interaction between Relish and *lncRNA-CR33942*, with the known interaction pair of *roX2* and msl-2 as a positive control (56, 57). The predicted interaction probability score of Relish and *lncRNA-CR33942* was nearly consistent with those of the interaction pairs of *roX2* and msl-2 (**Figure 3**). In addition, the results of RIP-qPCR experiments showed that the enrichment fold of Flag-Rel68 for lncRNA-CR33942 was approximately 60 times that of the control group, which further confirmed their interaction (**Figure 3**). Since the NF-κB transcription factor Relish mainly activates the transcription of AMPs, ChIP-qPCR and dual-luciferase reporter assays were performed to investigate how *lncRNA-CR33942* influences the transcriptional regulatory function of Relish. The results of ChIP-qPCR revealed that the enrichment fold on the promoter of *AttA* and *Dpt* using anti-Flag was nearly 40 times that of anti-IgG, whereas the enrichment fold was further significantly enhanced after *lncRNA-CR33942* overexpression (**Figure 3**). Similarly, the dual-luciferase reporter assay showed that Rel68 could promote *AttA* and *Dpt* promoter activity, which were further significantly enhanced after *lncRNA-CR33942* overexpression (**Figure 3**). In summary, these results suggest that *lncRNA-CR33942* interacts with Relish to promote its binding to the AMP promoter, thereby enhancing AMP expression.

### Relish Directly Activates the Transcription of *lncRNA-CR33942*

**Figure 4:**
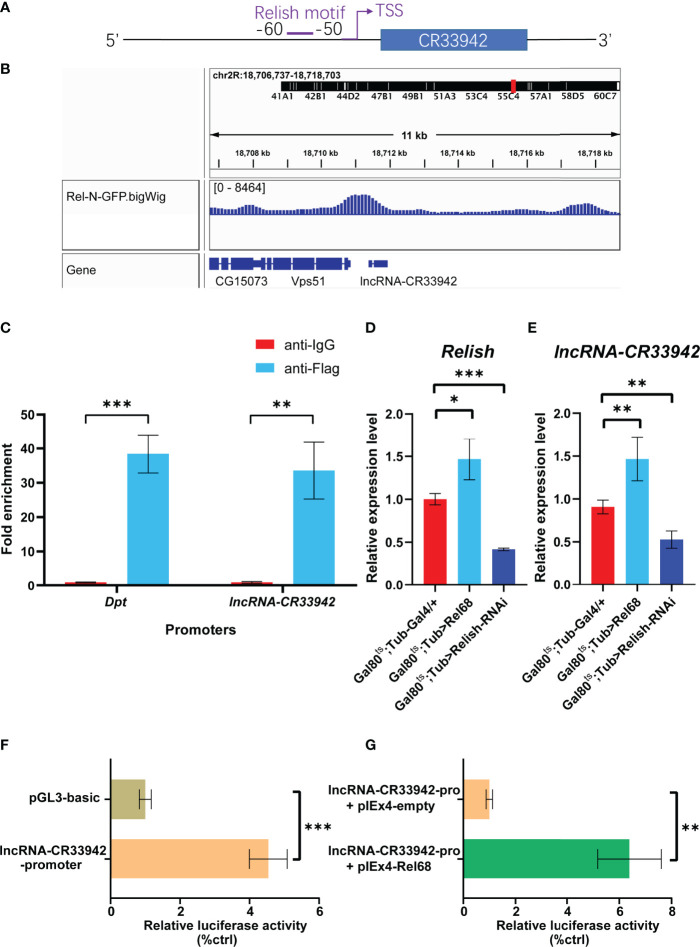
Relish directly activates the transcription of *lncRNA-CR33942*. **(A)** Schematic diagram of Relish motif in the lncRNA-CR33942 promoter region. **(B)** ChIP-seq visualization of Rel-N-GFP using IGV. **(C)** ChIP-qPCR was performed in Flag-Rel68 overexpressing S2 cells normalized to control expression levels. The expression levels of *Relish* **(D)** and *lncRNA-CR33942* **(E)** in Rel68 overexpressing and Rel68-RNAi flies. **(F, G)** The dual-luciferase reporter assays were performed to detect the activity of the *lncRNA-CR33942* promoter with or without Rel68 overexpression. *P < 0.05; **P < 0.01; ***P < 0.001.

To analyze the critical role of *lncRNA-CR33942* in the Imd pathway, we focused on the regulation of *lncRNA-CR33942* in the *Drosophila* Imd immune response. We found the motif of *Relish* at -60 to -50 bp upstream of the TSS site of *lncRNA-CR33942* using FIMO (https://meme-suite.org/meme/tools/fimo) and PROMO (http://alggen.lsi.upc.es/cgi-bin/promo_v3/promo/promoinit.cgi?dirDB=TF_8.3/) website with default parameters (**Figure 4**). In addition, we downloaded the ChIP-seq data of Rel-N-GFP from the ENCODE database (https://www.encodeproject.org/) and visualized the peak using IGV 2.9.4. An evident peak from the ChIP-seq of Rel-N-GFP was enriched in the promoter region of *lncRNA-CR33942* (**Figure 4**). To further confirm the authenticity of Relish binding to the lncRNA-CR33942 promoter, ChIP-qPCR was performed using S2 cells overexpressing Flag-Rel68. The results showed that the enrichment fold of the lncRNA-CR33942 promoter was approximately 30-fold higher than that of anti-IgG, which was close to the 40-fold enrichment fold of the positive control *Dpt* promoter (**Figure 4**). To investigate the function of Relish binding directly to the *lncRNA-CR33942* promoter, we examined the expression levels of *lncRNA-CR33942* in Rel68 overexpressing and Relish-RNAi flies using RT-qPCR. As expected, *lncRNA-CR33942* expression was significantly upregulated in Rel68-overexpressing flies and significantly downregulated in Relish-RNAi flies compared to the controls (**Figure E**). Furthermore, similar results were confirmed using dual-luciferase reporter assays. First, we cloned the *lncRNA-CR33942* promoter region into the pGL3-basic plasmid and detected its promoter activity (**Figure 4**). After Rel68 overexpression, the activity of the *lncRNA-CR33942* promoter significantly increased (**Figure 4**). These results indicated that Relish could directly bind to the *lncRNA-CR33942* promoter and promote its expression.

### Relish Promotes *lncRNA-CR33942* Transcription to Enhance Imd Immune Response

**Figure 5:**
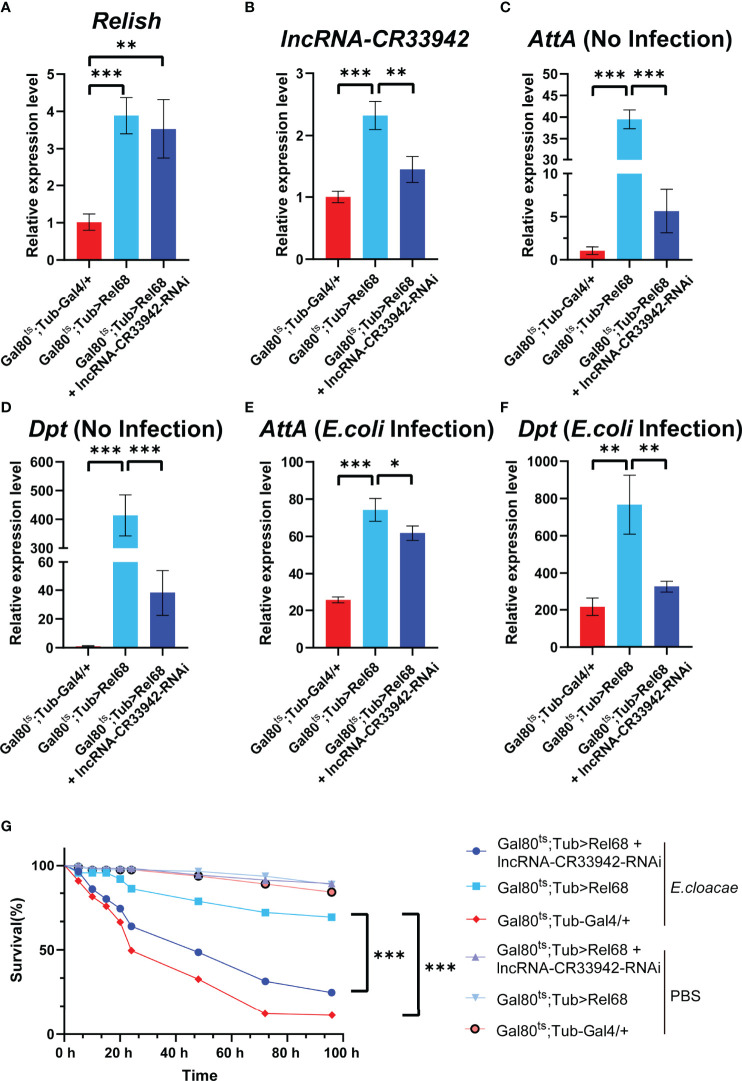
Relish promotes *lncRNA-CR33942* transcription to enhance Imd immune response. The expression levels of *Relish* **(A)** and *lncRNA-CR33942* **(B)** in Rel68 overexpressing and Rel68 + *lncRNA-CR33942*-RNAi co-overexpressing flies. *AttA* and *Dpt* expression levels in Rel68 overexpressing and Rel68 + *lncRNA-CR33942*-RNAi co-overexpressing flies with no infection **(C, D)** and 12 h post-infection **(E, F)**. **(G)** Changes in the survival rate of the Rel68 overexpressing, Rel68 + *lncRNA-CR33942*-RNAi co-overexpressing, and control flies were measured at 96 h after being treated with PBS or *Enterobacter cloacae. Gal80^ts^; Tub-Gal4/+* - PBS (n = 109), *Gal80^ts^; Tub> Rel68* - PBS (n = 107), *Gal80^ts^; Tub> Rel68 + lncRNA-CR33942-RNAi* - PBS (n = 104), *Gal80^ts^; Tub-Gal4/+* - *E. cloacae* (n = 107), *Gal80^ts^; Tub> Rel68* - *E. cloacae* (n = 109), *Gal80^ts^; Tub> Rel68 + lncRNA-CR33942-RNAi* - *E. cloacae* (n = 105). *P < 0.05; **P < 0.01; ***P < 0.001.

To determine whether Relish could promote the transcription of *lncRNA-CR33942* to regulate the Imd pathway *in vivo*, we constructed Rel68 overexpressing and Rel68 + *lncRNA-CR33942*-RNAi co-overexpressing flies and detected the expression of *Relish* and *lncRNA-CR33942* in these flies to ensure a successful construction (**Figure 5B**). The expression levels of *Dpt* and *AttA* were examined in Rel68 overexpressing, Rel68 + *lncRNA-CR33942*-RNAi co-overexpressing, and control flies at 12 h post-infection and no infection. In the absence of infection, *AttA* and *Dpt* expression levels were significantly upregulated approximately 40-fold and 400-fold, respectively, in Rel68-overexpressing flies compared with the controls, and significantly decreased in Rel68 + *lncRNA-CR33942*-RNAi co-overexpressing flies (**Figure 5D**). At 12 h after *E. coli* infection, the expression levels of both *AttA* and *Dpt* in Rel68 overexpressing flies were approximately 4-fold higher than those in controls, while the expression levels of *AttA* and *Dpt* in Rel68 + *lncRNA-CR33942*-RNAi co-overexpressing flies were significantly downregulated compared to those in Rel68 overexpressing flies (**Figure 5F**). Remarkably, we investigated the survival of these flies following septic injury by the lethal Gram-negative bacterium *E. cloacae*. Similar to the RT-qPCR results, the results showed that the survival rate of Rel68-overexpressing flies with the highest AMP levels was significantly prolonged. In contrast, the survival rate and AMP levels of Rel68 + *lncRNA*-*CR33942*-RNAi co-overexpressing flies were notably decreased compared with Rel68-overexpressing flies (**Figure 5**). Overall, these results suggested that Relish can activate *lncRNA-CR33942* transcription to enhance deficient immune responses and help extend the *Drosophila* survival rate.

### Relish/*lncRNA-CR33942*/AMPs Regulatory Axis Contributes to *Drosophila* Imd Immune Response

**Figure 6:**
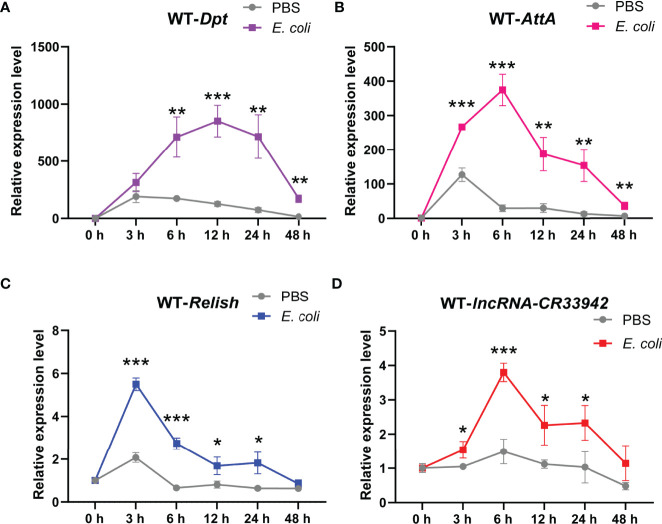
The Relish/*lncRNA-CR33942*/AMPs regulatory axis contributes to the *Drosophila* Imd immune response. The expression levels of *Dpt* **(A)**, *AttA* **(B)**, *Relish* **(C)**, and *lncRNA-CR33942* **(D)** in wild-type flies (*w^1118^*) at different time points (0, 3, 6, 12, 24, and 48 h) after *Escherichia coli* infection. *P < 0.05; **P < 0.01; ***P < 0.001.

To further explore the physiological function of the Relish/*lncRNA-CR33942*/AMPs regulatory axis, we monitored the dynamic expression of *Relish*, *lncRNA-CR33942*, *Dpt*, and *AttA* in wild-type flies (*w^1118^*) at different time points (0, 3, 6, 12, 24, and 48 h) after *E. coli* infection. The results showed that *Dpt* expression was significantly upregulated at 6 h after *E. coli* infection compared with the PBS-injected flies and reached a peak at 12 h, reaching approximately 1000-fold that of uninfected flies, and then decreased to close to the original level at 48 h (**Figure 6**). Similar to *Dpt*, the dynamic expression level of *AttA* was significantly increased at each time point after infection compared to the control, but the peak reached approximately 400 times that of uninfected cells at 6 h after infection (**Figure 6**). In contrast, the expression level of the transcription factor *Relish* had reached a peak at 3 h post infection and then decreased (**Figure 6**). The dynamic expression level of *lncRNA-CR33942* was similar to that of *AttA*, except that the peak at 6 h was four times that of the control (**Figure 6**).

**Figure 7:**
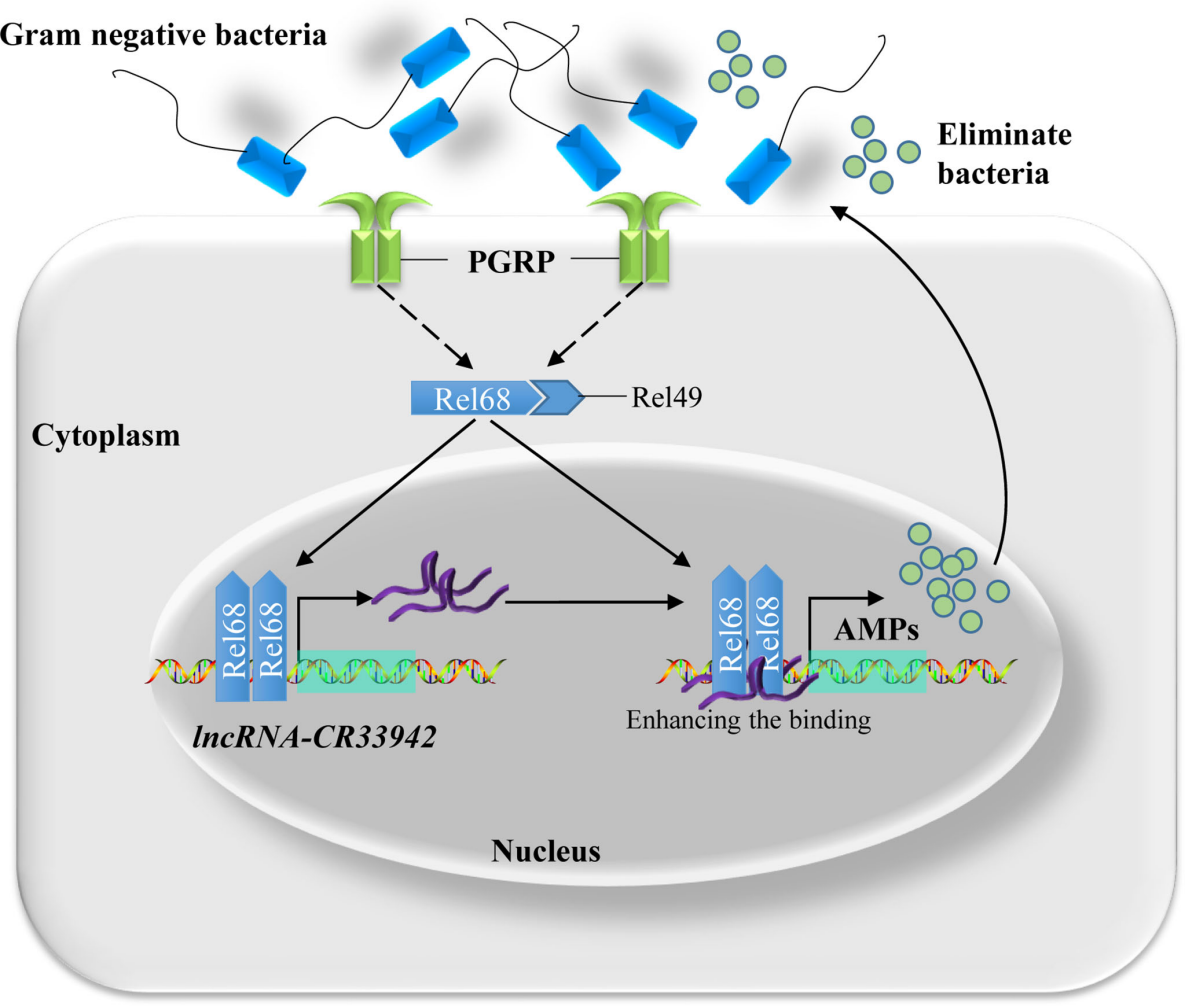
Schematic diagram of Relish-mediated *lncRNA-CR33942* enhancing *Drosophila* Imd immune response and maintaining immune homeostasis. Gram-negative bacteria activated the Imd signaling pathway, and transcription factor Relish entered the nucleus to promote the expression of AMPs and *lncRNA-CR33942*. The latter interacted with Relish and enhanced its binding to the AMP promoters, which in turn strengthen the insufficient immune response and maintaining immune homeostasis.

Based on the above results, we proposed a regulatory paradigm for the Relish/*lncRNA-CR33942*/AMP axis in response to the Imd pathway. First, upon attack by Gram-negative bacteria, the Imd pathway is activated, and Relish enters the nucleus to activate the transcription of AMPs and simultaneously promote the expression of *lncRNA-CR33942*. Next, *lncRNA-CR33942* would guide further binding of Relish to the AMP promoters, thereby enhancing the insufficient Imd immune response and maintaining immune homeostasis (**Figure 7**).

## Discussion

The duration and strength of innate immunity need to be tightly regulated because it can be detrimental to the host and can lead to death (58, 59). lncRNAs are a class of heavily transcribed RNAs but lack translatable ORFs and play important regulatory functions in innate immunity. For example, in the differentiation and development of immune cells, lncRNA-H19 (60), lncRNA-Xist (61), lncRNA-HSC1, and lncRNA-HSC2 (62) can regulate quiescence and self-renewal of hematopoietic stem cells. In addition, lncRNA-DC (63) and lncRNA-Morrbid (64) help differentiate into specific myeloid cells. However, the functions and mechanisms of lncRNAs in *Drosophila* innate immunity and their dynamic expression patterns are still poorly understood. In this study, we investigated how *lncRNA-CR33942* positively regulates the *Drosophila* Imd pathway and the dynamic regulatory mechanism of the relish/lncRNA-CR33942/AMP regulatory axis in Imd immune homeostasis. Together with previous study revealing the effect of *lncRNA-CR33942* on the Toll pathway, the regulator mechanism of *lncRNA-CR33942* in *Drosophila* innate immunity was further clarified (44). This further enriched our understanding of lncRNA regulation in *Drosophila* immune homeostasis.

*LncRNA-CR33942*, as an intergenic lncRNA, is located beside the protein-coding gene *Vps51* (genomic loci 2R:18,711,391.18,711,951 [+]) and has not been well studied to date. We found the data in FlyAtlas showed that the most abundant distribution site of *lncRNA-CR33942* was the fat body of larvae and adults, which is a crucial immune organ in *Drosophila* (65). In addition, upregulated DEGs in *lncRNA-CR33942*-overexpressing flies after infection were only significantly enriched in the Toll and Imd signaling pathways, and *lncRNA-CR33942* was positively correlated with AMP expression (**Figures 1, 2C**). These results confirmed that *lncRNA-CR33942* positively regulates the *Drosophila* Imd immune response. Notably, only lncRNA-VINR has been reported to regulate the *Drosophila* Imd pathway AMPs (41). IBIN, which is upregulated several hundred-fold upon *M. luteus* infection and is thought to regulate innate immunity and metabolism but was later identified as an encoding gene (66, 67). In contrast, the expression levels of *lncRNA-CR33942* increased several-fold after infection with *E. coli*, unlike the hundred-fold increase in AMPs (**Figure 6**). This suggests that *lncRNA-CR33942* is more likely to function as a regulator than as an effector.

The *Drosophila* Toll and Imd signaling pathways are crucial humoral immune pathways against Gram-positive bacterial/fungal and Gram-negative bacterial invasion, respectively, and are highly conserved with the mammalian TLR and TNFR signaling pathways (2, 68, 69). Our results implied that *lncRNA-CR33942* could regulate both pathways and influence various AMPs in response to different pathogens. Notably, the regulation of different AMPs by *lncRNA-CR33942* was different. For example, the expression level of *AttA* was decreased by hundreds of times in infected *lncRNA-CR33942*-RNAi flies and was increased two-fold in *lncRNA-CR33942*-overexpressing flies, whereas *Dpt* was fine-tuned (**Figure 1**). We speculated that the different regulatory functions of *lncRNA-CR33942* on different AMPs may be because *lncRNA-CR33942* affects the binding ability of Relish to the AMP promoter. Although the Toll and Imd pathways respond to different pathogens, some of their components, such as the NF-κB transcription factors Dif/Dorsal and Relish, are highly homologous. Therefore, mechanistically, *lncRNA-CR33942* can interact with the NF-κB transcription factors of both pathways to promote AMP transcription, thereby enhancing the *Drosophila* Toll and Imd pathways in response to the invasion of various pathogens.

NF-κB is one of the most important transcription factors in the immune response and understanding how NF-κB regulates lncRNAs can reveal the dynamic regulatory mechanism of lncRNAs in immune processes and their important role in promoting immune homeostasis. However, regulation of lncRNA transcription by NF-κB has mainly been studied in mammals. For example, NF-κB promotes the expression of lncRNA-FIRRE to regulate expression of inflammatory genes (70). In addition, NF-κB-induced lincRNA-Cox2 acts as a co-activator of NF-κB to regulate late-stage inflammatory genes in macrophages (71). However, most of these studies were from immune cell lines, and systematic studies *in vivo* were lacking. Therefore, we systematically explored the immune regulatory axis of Relish/*lncRNA-CR33942*/AMPs in *Drosophila*, which broadens our understanding of innate immune regulation and maintenance of homeostasis.

In conclusion, we revealed the mechanism by which the NF-κB transcription factor Relish-induced *lncRNA-CR33942* regulates Imd immune responses and maintains immune homeostasis. Briefly, once invaded by Gram-negative bacteria, the Imd pathway is activated, and Relish is cleaved into the nucleus to facilitate *lncRNA-CR33942* transcription. *lncRNA-CR33942* further interacted with Relish to enhance the binding of Relish to AMP promoters, thereby enhancing the *Drosophila* Imd immune response and maintaining immune homeostasis (**Figure 7**). Our study not only discovered a novel Relish/*lncRNA-CR33942*/AMP regulatory axis, but also has important guiding significance for elucidating the complex regulatory mechanism of the innate immune response in animals.

## Data Availability Statement

The datasets presented in this study can be found in online repositories. The names of the repository/repositories and accession number(s) can be found below: https://www.ncbi.nlm.nih.gov/geo/query/acc.cgi?acc=GSE198991.

## Author Contributions

HZ, SW, RL, SL, and PJ were mainly responsible for experimental design. HZ, SW, and LL were responsible for experiment implementation and data analysis. HZ and PJ wrote the article. RL, SL, and PJ were responsible for providing fund support. All authors contributed to the article and approved the submitted version.

## Funding

This work was supported by the National Natural Science Youth Foundation of China (No. 32100390), the National Natural Science Youth Foundation of China (No. 31802015), and a project funded by the Priority Academic Program Development of Jiangsu Higher Education Institute.

## Conflict of Interest

The authors declare that the research was conducted in the absence of any commercial or financial relationships that could be construed as a potential conflict of interest.

## Publisher’s Note

All claims expressed in this article are solely those of the authors and do not necessarily represent those of their affiliated organizations, or those of the publisher, the editors and the reviewers. Any product that may be evaluated in this article, or claim that may be made by its manufacturer, is not guaranteed or endorsed by the publisher.
