# Supplementary material for: Drosophila Relish Activating lncRNA-CR33942 Transcription Facilitates Antimicrobial Peptide Expression in Imd Innate Immune Response
Source: Front Immunol. 2022 Jun 2;13:905899. doi: 10.3389/fimmu.2022.905899 (PMC9201911; doi:10.3389/fimmu.2022.905899)
Supplement: Supplementary file 1 [file Table_1.docx]

**Supplementary Table 1. Primers used in the study:**

| Name | Primer sequence(5’ – 3’) |
| --- | --- |
| rp49-qF | GACGCTTCAAGGGACAGTATCTG |
| rp49-qR | AAACGCGGTTCTGCATGAG |
| ChIP-Dpt-qF | TCCCCTGGTGGTATTTGTTTT |
| ChIP-Dpt-qR | TGCTCTTTTATAGGCCGCTTT |
| ChIP-AttA-qF | GCGGCGAGGGTGAAACT |
| ChIP-AttA-qR | GATGCCTTATCAAAGCGAAATT |
| AttA-qF | GACACAATCTGGATGCCAAG |
| AttA-qR | AATCCAGACCAGCTCCATTC |
| Dpt-qF | TCCGATGCCCGACGACATGA |
| Dpt-qR | TGGCGTCCATTGTCGCTGGT |
| ChIP-lncRNA-CR33942-qF | TCGTGAGAGACGTGAACTGAGA |
| ChIP-lncRNA-CR33942-qR | AGCTGCAATTAATGCGGAAA |
| pGL3-lncRNA-CR33942-pro-F | AGGTACCAAACCAGCGACAGACGAAC |
| pGL3-lncRNA-CR33942-pro-R | CAGATCTGCGGAAACAAAGAGTAATAGC |
| CR33942-qF | GTGACTGCTGATTGCTTAA |
| CR33942-qR | GGAGACTGTTGAATTACTTAGG |
